# The Rules of the Game: Towards a Theory of Practice for Performance Nutritionists in Professional Soccer Using Bourdieu’s Concepts of Habitus, Capital and Field

**DOI:** 10.1007/s40279-026-02408-5

**Published:** 2026-03-08

**Authors:** Wee Lun Foo, Colum J. Cronin, Graeme L. Close, James P. Morton

**Affiliations:** 1 Research Institute for Sport and Exercise Sciences (RISES), Liverpool John Moores University, Liverpool, UK; 2 Tottenham Hotspur Football Club, Enfield, London, UK

## Abstract

**Background and Objective:**

Performance nutrition is now an established discipline in professional soccer; however, limited knowledge exists on how practitioners can navigate through the unique cultural environment of the men’s professional game. Accordingly, we explored the perspectives of players and stakeholders from the English Premier League on the attributes they perceive to underpin successful performance nutrition practice.

**Methods:**

Guided by an interpretivist paradigm with a critical perspective (recognising that reality is also subjectively and socially constructed), qualitative face-to-face semi-structured interviews were conducted with purposively sampled English Premier League stakeholders from August 2024 to December 2024, including players (*n* = 4), coaches (*n* = 4), sports scientists (*n* = 2), physiotherapists (*n* = 2), a chef (*n* = 1) and a medical doctor (n = 1). Data were abductively analysed using a thematic analysis informed by Bourdieu’s concepts of habitus, capital and field.

**Results:**

Three key themes were identified. It is perceived that (1) Field: successful performance nutritionists must recognise and adapt to the hierarchical structure, entrenched cultural norms and doxic practices of professional soccer, adapting their strategies to gain support from coaches, players and staff; (2) Capital: effective performance nutrition practice requires strategic deployment of cultural capital (technical, sports-specific and interdisciplinary knowledge) and social capital (ability to build trust and relationships with key stakeholders) to establish credibility and influence practice; (3) Habitus: the ability to accumulate and mobilise these forms of capital is underpinned by a habitus congruent with elite soccer’s culture, characterised by passion, resilience, adaptability and positivity.

**Conclusions:**

These data provides a comprehensive interpretation of the unwritten rules of professional soccer, demonstrating that the perceived success for performance nutritionists in the English Premier League extends beyond technical expertise, requiring the ability to navigate tacit field rules, strategically mobilise cultural and social capital and embody a habitus aligned with the values of elite soccer through passion, adaptability, positivity and resilience.

## Key Points

**Table:** 

The professional soccer field operates as a hierarchical social arena governed by its own written and unwritten rules, where doxic authority of coaches, the elevated status of players and entrenched beliefs and norms (e.g. rigid expectations around body composition) shape the practices and autonomy of performance nutritionists.
To successfully navigate these implicit “rules of the game”, performance nutritionists must strategically deploy their cultural capital, encompassing technical, sport-specific and interdisciplinary expertise, together with social capital, including the ability to build trust and maintain strong relationships with key stakeholders, all supported by a habitus defined by passion, positivity, adaptability and resilience.
Expectations for performance nutritionists to conform to entrenched hierarchies and doxic norms to consistently “go above and beyond” may reinforce unsustainable working practices, potentially affect service quality and highlight the importance of adequate staffing to support a sustainable and high-quality performance nutrition service.

## Introduction

The role of performance nutrition in professional soccer has developed markedly over recent decades. Once regarded with scepticism and largely confined to medical staff providing basic dietary advice [1], nutrition support has progressively evolved through part-time consultancy models to the appointment of dedicated specialists embedded within multidisciplinary teams in elite soccer clubs [2, 3]. This developing profession has been driven by increasing economic investment in the game [3–5] and is supported by a growing body of applied research [6], which has elevated the cultural capital of performance nutrition within the professional soccer field. However, progress towards professionalism has largely been confined to the top tier of the soccer pyramid, with further improvements required across lower-league teams and academies [3]. The publication of documents such as the UEFA Expert Group Statement on Nutrition in 2021 [7] further reflects this evolution, establishing nutrition as a recognised and integral component of elite soccer performance. Nonetheless, while the physiological basis of performance nutrition for soccer is increasingly well defined, much less is known about how performance nutritionists themselves navigate the social, cultural and organisational structures of professional soccer to deliver their practice.

Professional soccer represents a distinctive social arena with its own internal logic, hierarchies of power and deeply embedded cultural norms. Within this field, multiple key stakeholders including players, coaches, performance and medical staff interact in ways that shape both the opportunities and barriers for performance nutrition practice, with each agent bringing different priorities, values and forms of capital that influence decision making and the day-to-day work of performance nutritionists [3, 8, 9]. Coaches and managers, in particular, often occupy a dominant position, with their authority extending into domains such as nutrition provision and body composition management, thereby exerting a direct influence on how nutrition support is delivered at the club level [3]. At times, this hierarchical dynamic can create tensions, as nutritionists may feel pressured to adopt practices that conflict with their professional values, potentially undermining their credibility and straining the nutritionist–player relationship [9]. For instance, the imposition of arbitrary body mass or composition targets by coaching staff, a practice widely reported in professional soccer [3], can encourage harmful dietary behaviours and body image concerns among players [9, 10]. Despite increasing professionalism in soccer, these challenges persist, and there remains a paucity of research examining what it takes for performance nutritionists to be effective within this highly complex and culturally specific environment [11].

To address these challenges, Bourdieu’s theory of practice [12] provides a valuable lens for understanding what underpins the effectiveness of performance nutritionists working in professional soccer. Through the interrelated concepts of habitus, capital and field, it highlights how individual dispositions and resources interact with the structured environment of professional soccer. Habitus reflects the embodied values and practices shaped by prior experiences and education, thus influencing how nutritionists interpret and respond to the challenges of the professional soccer environment [13]. Capital, including cultural (scientific expertise, qualifications), social (relationships, trust, networks) or symbolic (status, legitimacy, recognition), shapes their capacity to exert influence within the club hierarchy [14], while field represents the environment itself, defined by power relationships, hierarchies and taken-for-granted beliefs (doxa). Although effective practitioners are expected to demonstrate adaptability, empathy, trustworthiness and strong technical expertise [11], research suggests that the more decisive attribute is the ability to appreciate the unique culture of the sport and build meaningful relationships with players and coaches [15].

Using this theoretical framework, the aim of this study is to qualitatively explore the perspectives of professional players and key stakeholders on their perceptions of what defines a successful performance nutritionist in the English Premier League (EPL). Drawing on the views of players, coaches, sport science and medical staff and chefs, this study provides novel insights into the qualities, skills and forms of capital that are regarded as most valuable within the distinctive cultural context of elite soccer. In doing so, we aimed to characterise a “theory of practice” that may offer practical guidance for performance nutritionists working in the elite soccer environment.

## Methods

### Research Philosophy and Positionality

Many nutritionists, particularly those working in performance contexts, are well aware of the importance of evidence-based science-backed practices. Nevertheless, a range of other factors can influence how each practitioner applies this knowledge in real-world settings. This study was underpinned by an interpretivist paradigm, which recognises that for individuals involved in nutrition provision, practice is socially constructed, context dependent and shaped by individual experiences [16]. Aligned with a relativist ontology, this perspective assumes the existence of multiple coexisting ways of being a nutritionist, shaped by social, cultural and historical contexts of those involved [17]. On this basis, knowledge is not objectively discovered but is co-constructed through inquisitive interaction. These propositions make interpretivism particularly well suited to explore the nuanced practices of sports nutritionists operating within context-specific elite soccer environments. Additionally, the researchers adopted a self-critical perspective on their positionality. While interpretivism seeks to understand how individuals make sense of their world, the critical paradigm extends this by interrogating the power relationships and dominant ideologies that shape those meanings [18]. This criticality recognises the non-neutral role of the researchers, whose positions within the professional soccer context necessitates reflexivity and an awareness of how their own capital and habitus influence the research process. This dual-orientation supports a rich and critical understanding of how performance nutrition is practiced, legitimised and negotiated within the power-laden field of professional soccer. Building upon this philosophical foundation, we conducted a qualitative study to explore the experiences and perceptions of key stakeholders operating within the complex social landscape of elite soccer. Guided by interpretivist and critical perspectives our sampling, data collection and analysis procedures were designed to produce a trustworthy and transparent account of the theory of practice underpinning the perception of what it means to be a successful sports nutritionist within EPL. This study adhered to the Standard for Reporting Qualitative Research [19] to ensure rigour, clarity and methodological transparency throughout the research process.

### Participants

To develop a comprehensive understanding of the qualities that contribute to the perceived success of a performance nutritionist in professional soccer, a purposive sampling strategy was employed to recruit key stakeholders from a single EPL club. A total of 14 participants were recruited and interviewed from August 2024 to December 2024, including coaches (*n* = 4), players (*n* = 4), physiotherapists (*n* = 2), sports scientists (*n* = 2), a medical doctor (*n* = 1) and a club chef (*n* = 1). This methodology mirrors previous qualitative inquiries into nutrition culture within the professional soccer domain [3], where the objective is not to generalise to a wider population (e.g. other leagues, sports) but to gain rich contextualised insights from a deliberately chosen expert and hard-to-reach sample. While all participants were contracted to a single club at the point of data collection, their narratives reflected cumulative professional experiences across a range of teams and leagues including the EPL, Serie A, Bundesliga, Ligue 1, Turkish Süper Lig, Australian A-League, Scottish Premiership and Liga Portugal. In line with qualitative research conventions, the sample size was not predetermined but guided by the data analysis process, with recruitment ceasing once a diverse range of perspectives and sufficient thematic depth had been achieved [20, 21]. Ethical approval was obtained from the Liverpool John Moores University Research Ethics Committee and in accordance with this approval, further participant details are withheld to ensure confidentiality. All participants provided verbal and written informed consent prior to taking part in the interviews.

### Procedures

All 14 participants engaged in semi-structured interviews (mean: 23 min; range 9–48 min), with an “open-ended” approach [22], where questions were posed in a conversational and informal manner to encourage voluntary input and detailed responses [23]. The questions (see Tables 1, 2, 3) were informed by the study aims and Bourdieu’s concepts of habitus, capital, field and doxa practices. For instance, initial questions were neutrally framed such as ‘What are your thoughts on …?’. Subsequently, probing questions were used to elicit further insights [24]. This format of enquiry enabled participants the freedom to express their experiences and opinions and to guide the discussion toward areas they deemed significant [25]. Consequently, the study’s findings extend beyond the scope of the participants’ current club, providing a comprehensive understanding of their involvement with previous teams across different soccer leagues. To assess the suitability of the interview questions, pilot interviews were conducted with two support staff (one sports scientist and one medical doctor) from the same club. Based on the feedback from the senior co-authors (CJC and JPM) for these pilot interviews, the wording of some questions was revised. Pilot interviews were not included in the analysis. All interviews took place in a private office at the club’s training facility and were audio-recorded, then transcribed verbatim. The interviewer was well versed in the professional soccer subculture, having worked as a performance nutritionist in the industry for the past 5 years. While this familiarity could potentially bias the interviewer’s approach, it was considered advantageous because of their fluency in understanding the key stakeholders’ jargon and informal language and their ability to develop rapport with participants [26]. To prevent leading questions, strategies such as piloting interview questions and utilising open-ended questions were implemented.

**Table 1:** Interview guide and aims for performance and non-performance staff

Interview questions	Prompts	Aims	
*Domain 1: Participant’s background and experiences working with performance nutritionists*			
Q1: Can you provide a brief overview of your career up to this point? Q2: Can you tell me more about your experience working with a nutritionist? Q3: In your experience working with a performance nutritionist, how would you describe their role? Q4: How has the role of nutritionist evolved over time?	F1: When did you first start in your current position? F2: When did you first start working with a nutritionist? How did you feel? F3: What are their duties and responsibilities? F4: What drives these changes?	A1: To acquire understanding about the prior professional background of participants A2: To understand participants’ prior experiences working with performance nutritionists A3: To understand participants’ perceived roles and responsibilities of performance nutritionists A4: To understand shifts in responsibilities, approaches or areas of focus that nutritionists have undergone	
*Domain 2: Factors that determine the success of a performance nutritionist in soccer*			
Q1 Based on your experience, what qualities and characteristics have you observed in a nutritionist who has had a positive impact within your multidisciplinary team (MDT)? Q2: What specific outcomes or improvements have you observed when working with a performance nutritionist? Q3: Based on your experience what qualities and characteristics have you observed in a nutritionist who has NOT had a positive impact within your MDT? Q4: What are your experiences with body composition in soccer?	F1: Can you share an example of successful intervention or strategy that a performance nutritionist has implemented? F2: How has nutrition improved players’ performance and development? Can nutrition make an impact on team selection? F3: What challenges have you encountered while working with a performance nutritionist? How are they addressed? F4: Where has this come from? Who is involved? How has body composition influenced selection?		A1: To identify specific qualities and characteristics of a nutritionist that contribute to their effectiveness and positive impact within an MDT A2: To gather concrete examples of the positive results or changes that have occurred as a result of working with a performance nutritionist A3: To identify and understand the traits or behaviours of a nutritionist that may hinder their effectiveness within an MDT A4: To gather insights on how body composition is managed and perceived in professional soccer
*Domain 3: Future directions of performance nutritionists*			
Q1: How do you think performance nutritionists could improve? Q2: How do you foresee the role of performance nutritionists changing in the future?	F1: What is missing from their service provision? Skills? Knowledge? F2: Why do you think that?		A1: To identify the areas in performance nutrition that participants believe need improvement A2: To investigate participants’ perspectives on the anticipated shifts in the role of nutritionists in the future

**Table 2:** Interview guide and aims for coaches

Interview questions	Prompts	Aims	
*Domain 1: Participant’s background and experiences working with performance nutritionists*			
Q1: In your career so far, can you tell me more about your experience working with nutritionists? Q2: In your experience working with a performance nutritionist, how would you describe their role? Q3: How has the role of nutritionist evolved over time?	F1: When did you first start working with a nutritionist? How did they make you feel? F2: What are their duties and responsibilities? F3: What drives these changes?	A1: To understand participants’ prior experiences working with performance nutritionists A2: To understand participants’ perceived roles and responsibilities of performance nutritionists A3: To understand shifts in responsibilities, approaches or areas of focus that nutritionists have undergone	
*Domain 2: Factors that determine the success of a performance nutritionist in soccer*			
Q1: From your experience, what qualities or characteristics have you observed in a nutritionist who has positively influenced the player you coach? Q2: What specific outcomes or improvements have you observed when working with a performance nutritionist? Q3: From your experience, what qualities or characteristics have you observed in a nutritionist who has NOT positively influenced the player you coach? Q4: What are your experiences with body composition in soccer?	F1: Can you share an example of successful intervention or strategy that a performance nutritionist has implemented? F2: How has nutrition improved players’ performance and development? Can nutrition make an impact on team selection? F3: What challenges have you encountered while working with a performance nutritionist? How are they addressed? F4: Where has this come from? Who is involved? How has body composition influenced selection?		A1: To identify specific qualities and characteristics of a nutritionist that contribute to their effectiveness and positive impact within an MDT A2: To gather concrete examples of the positive results or changes that have occurred as a result of working with a performance nutritionist A3: To identify and understand the traits or behaviours of a nutritionist that may hinder their effectiveness within an MDT A4: To gather insights on how body composition is managed and perceived in professional soccer
*Domain 3: Future directions of performance nutritionists*			
Q1: How do you think performance nutritionists could improve? Q2: How do you foresee the role of performance nutritionists changing in the future?	F1: What is missing from their service provision? Skills? Knowledge? F2: Why do you think that?		A1: To identify the areas in performance nutrition that participants believe need improvement A2: To investigate participants’ perspectives on the anticipated shifts in the role of nutritionists in the future

**Table 3:** Interview guide and aims for players

Interview questions	Prompts	Aims	
*Domain 1: Participant’s background and experiences working with performance nutritionists*			
Q1: Have you worked with a nutritionist before? Q2: In your experience working with a performance nutritionist, how would you describe their role? Q3: How has the role of a nutritionist evolved over time?	F1: When did you first start working with a nutritionist? F2: What are their duties and responsibilities? F3: What drives these changes?	A1: To understand participants’ prior experiences working with performance nutritionists A2: To understand participants’ perceived roles and responsibilities of performance nutritionists A3: To understand shifts in responsibilities, approaches or areas of focus that nutritionists have undergone	
*Domain 2: Factors that determine the success of a performance nutritionist in soccer*			
Q1: From your experience, what qualities or characteristics have you observed in a nutritionist who has had a positive impact whilst working with you? Q2: What specific outcomes or improvements have you observed when working with a performance nutritionist? Q3: From your experience, what qualities or characteristics have you observed in a nutritionist who has NOT had a positive impact whilst working with you? Q4: What are your experiences with body composition in soccer?	F1: Can you share an example of a successful intervention or strategy that a performance nutritionist has implemented? F2: How has nutrition improved players’ performance and development? Can nutrition make an impact on team selection? F3: What challenges have you encountered while working with a performance nutritionist? How are they addressed? F4: Where has this come from? Who is involved? How has body composition influenced selection?		A1: To identify specific qualities and characteristics of a nutritionist that contribute to their effectiveness and positive impact within an MDT A2: To gather concrete examples of the positive results or changes that have occurred as a result of working with a performance nutritionist A3: To identify and understand the traits or behaviours of a nutritionist that may hinder their effectiveness within an MDT A4: To gather insights on how body composition is managed and perceived in professional soccer
*Domain 3: Future directions of performance nutritionists*			
Q1: How do you think performance nutritionists could improve? Q2: How do you foresee the role of performance nutritionists changing in the future?	F1: What is missing from their service provision? Skills? Knowledge? F2: Why do you think that?		A1: To identify the areas in performance nutrition that participants believe need improvement A2: To investigate participants’ perspectives on the anticipated shifts in the role of nutritionists in the future

### Data Analysis

All interviews were transcribed by the principal investigator. Data were analysed using an abductive approach, combining with inductive and deductive processes, while acknowledging the interpretative creativity involved in applying a theoretical framework to participants’ experiences [20]. A thematic analysis followed a six-stage process [27]: (1) familiarisation and immersion of the data through repeated reading and listening during transcription; (2) systematic initial coding process to identify relevant content; (3) reassessment of initial codes to identify patterns and generate preliminary themes aligned with the theoretical framework; (4) review of themes for coherence against the raw data; (5) refinement, definition and naming of themes once a consensus was reached; and (6) selection of data excerpts from each theme to present a concise, coherent and engaging narrative that reflects the data’s story within and across themes. To enhance rigour, the final author, unacquainted with the club and not involved in the interview process, acted as a “critical friend”, who independently checked and challenged the data analysis, theme generation and presentation of selected quotes [28]. The role of the critical friend is not to seek agreement or consensus but to encourage reflexivity by questioning interpretations and constructions of knowledge [29]. While the lead author’s role within the club and personal interest in the topic inevitably introduced subjectivity, this insider perspective was considered advantageous for contextual understanding and rapport building. The involvement of the critical friend, who interrogated and challenged the analysis, helped ensure a balance between insider insight and analytic rigour [30].

### Methodological Trustworthiness and Rigour

To ensure rigour, several measures consistent with qualitative methods and interpretivist paradigms were employed [31]. These included recruiting a diverse sample, applying a robust theoretical framework and piloting the interview questions. Independent members of the research team, separate from the lead author, provided critical feedback on the interview techniques and data analysis process. Rigour was further reinforced through open discussions among all authors, who acted as critical friends and maintained a reflective stance throughout [31]. The worthiness of this research topic was justified by addressing the gap in evidence and practice within this population [32, 33]. Furthermore, the results and discussion section outlines three themes, supported by quotations from the data, enabling readers to interpret the findings independently and consider the applicability to their own circumstances [28]. To improve the credibility of the manuscript, member reflections were conducted by providing participants with a one-page summary of our interpretations and findings for their feedback [34].

## Results and Discussion

**Fig. 1:**
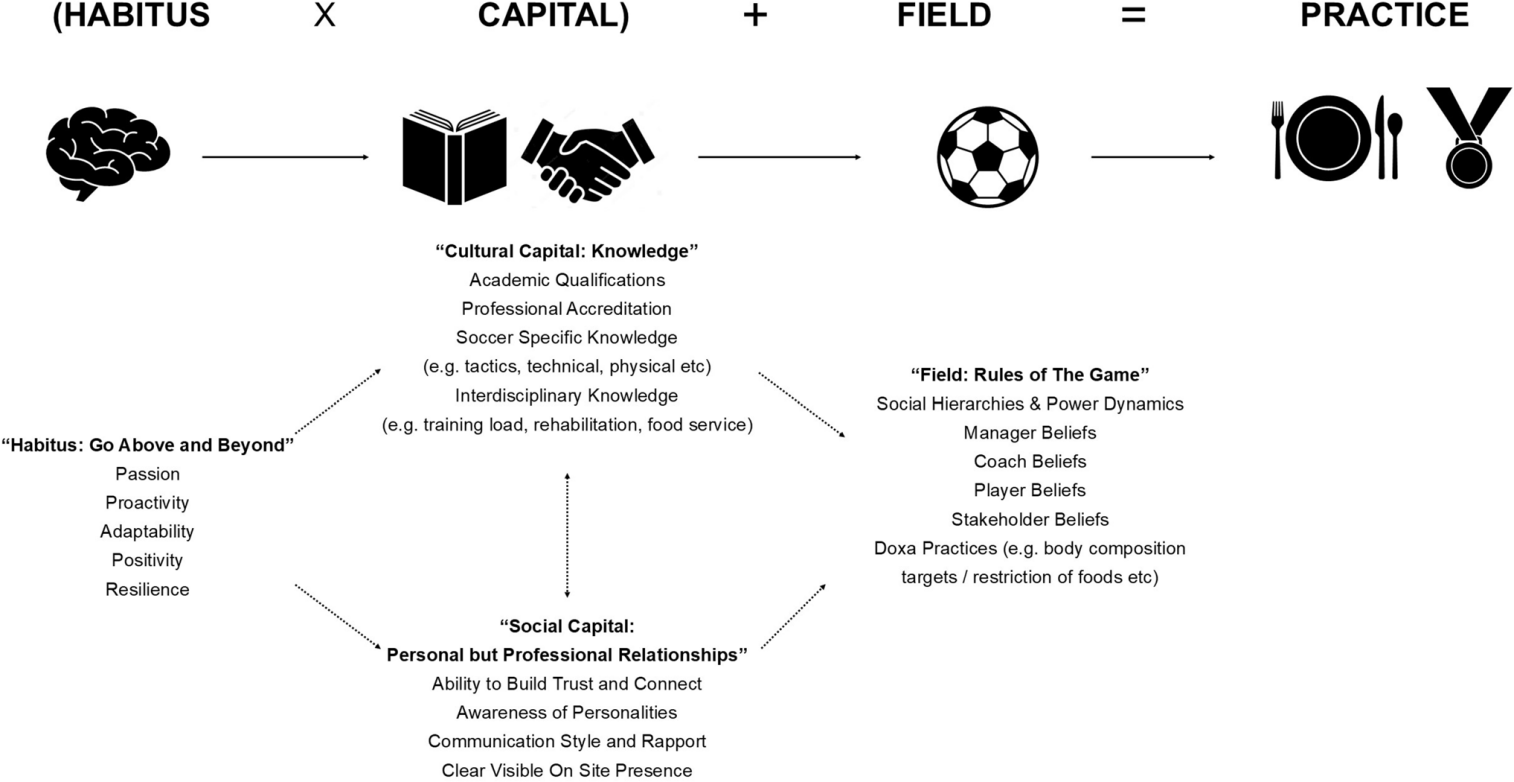
Towards a theory of practice for performance nutrition in professional soccer. How practitioners practice (hence the nature of practice and associated service provision) is dependent on their habitus, accumulated capitals and how they navigate and present themselves within a given field.  In this context, a habitus that is characterised by the ability to go above and beyond is more likely to increase the accumulation of both cultural capital and social capitals. In turn, both habitus and capitals can subsequently determine a practitioner’s ability to understand the rules of the game, thereby informing how they navigate the social hierarchies that exist within the field (i.e. survive and thrive). In considering this theory of practice, it is noteworthy that a specific nutritionist may practice differently (and indeed be perceived to have different success) according to whichever field they are working in. For example, how one practices within a soccer environment may look and feel very different to how they would practice within other team sports (e.g. rugby, basketball etc), endurance sports (e.g. running or cycling etc) or weight sensitive sports (e.g. combat sports, gymnastics etc). Furthermore, to be perceived as successful in each of these sports (according to the relevant athletes, coaches and stakeholders), nutritionists may need therefore to present with a different habitus, accumulated capitals and an appreciation of the rules of the game (i.e. the cultural nuances and social dynamics within a given sporting field)

Using a reflexive thematic analysis approach, we identified three themes based on the concepts of field, capital and habitus: (1) Field: a successful performance nutritionist must understand the rules of the game, (2) Capital: a successful performance nutritionist must have sufficient technical knowledge (cultural capital) but also the ability to build relationships (social capital) and (3) Habitus: the success of a nutritionist is dependent on their ability to accumulate capital and is shaped by their habitus. Together, these themes illustrate how successful performance nutritionists must have the ability to navigate challenges in the field of professional soccer, build and accumulate different forms of capital and align their habitus to the changing environment of professional soccer. Aligned with previous qualitative research conducted in elite soccer settings [3], these themes are further critically examined within the discussion section, providing a nuanced exploration of each theme in relation to the existing literature. Figure 1 illustrates the interrelationship between the three themes and their collective contribution to developing a theory of practice for performance nutrition in professional soccer.

### Field: A Successful Performance Nutritionist Must Understand the Rules of the Game

The professional soccer environment operates as a distinct social field, characterised by its own internal logic and established structures of practice [35]. Within this field, various positions are occupied by key stakeholders, including coaches, players and members of the sports science and medical teams. Across the participants’ experiences at various clubs, the professional soccer field is deemed to be inherently hierarchical; previous research has demonstrated that coaches hold a dominant position of influence, with their decisions significantly shaping the dietary practices of professional players [1, 3]. Consequently, for performance nutritionists to be effective within this context, it is essential to first understand the manager’s perspective on nutrition. As Participant 14 (coach) explained:

“The first bit of advice is I think you always need to understand where the managers are. So maybe you have a manager that don’t value it so then it’s harder for you to get really strong. If you have a manager that really values nutrition, then I think it’s easier to go really strong because you have the backing of the manager.”

Furthermore, players are widely regarded as the most valuable assets of a club [36]. Beyond their on-field contributions, they are frequently recognised as global icons who significantly contribute to the club’s public image and commercial interests [37]. Consequently, interactions between practitioners and players are shaped by the players’ elevated status within the club’s social hierarchy. Owing to their considerable symbolic capital, it is not uncommon for professional soccer players to exhibit a strong sense of self-assurance or what may be perceived as a heightened ego, which can present challenges when delivering feedback. Adopting rigid or authoritative communication styles may be counterproductive, as illustrated by Participant 12 (player):

“I think players have very big ego. All sportsmen do, because that’s a little bit what you need in soccer. If you guys talk bad to a player or tell him like “Hey, you have to do this!” then it’s going to have the opposite effect because the players will say “No, I will go my way! I will not do what you say, as you are not a soccer player. I will not listen to you, as I am the soccer player” and they will go their way. So, I think it’s you got to be very like “Here’s something you can try but if you don’t want it, then you don’t want it”.

Similarly, Participants 1 (chef) and 2 (physiotherapist) reinforced the importance of practitioners developing a nuanced understanding of the unique cultural dynamics within the field of professional soccer. They highlighted the potential challenges that can arise when practitioners fail to appreciate the distinct mindset and social norms embedded within the elite soccer environment:

**[Participant 1, Chef]** “We basically worked on trying to introduce Mediterranean diet. At that time, we have 19 nationalities and it didn’t appeal to a lot of them. We were between a rock and a hard place, trying to introduce things that not necessarily some of the players would have eaten.”

**[Participant 2, Physiotherapist]** “I think if that person doesn’t have an understanding of the environment. If that individual, I’m thinking of other staff that I might have seen in this environment that comes from other sporting environments, they tried to make nutritional strategies that worked in other environment that might have involved individual sports and individual athletes. Because of the mindset of a soccer player and an athlete is very different, so I think you have to understand the environment to try to get that buy-in with the players.”

Within any social field, the concept of doxa refers to the taken-for-granted beliefs, norms and practices that are deeply embedded within the collective consciousness of its members. These implicit forms of social knowledge function to uphold and reproduce existing power relationships, often without being openly questioned [12]. In professional soccer, doxic practices are particularly influential, shaping behaviours and expectations across all levels of the sports. A prominent example is the doxic beliefs surrounding the ideal body composition for professional soccer players, an assumption that has become normalised within soccer culture [3]. This was echoed by Participant 11 (player), who commented on the entrenched nature of body composition monitoring within the sport:

“It [body composition measurement] was ingrained in soccer, it’s probably the easiest marker to see whether someone is in shape. I don’t know where it comes from, but there probably is an obsession with weight and with body composition within the game. I don’t know where it comes from, maybe historically from coaches where in the past they probably didn’t have great diagnosis. So, they just decided, okay, the easiest one to jump onto is body fat."

Practitioners must exercise considerable caution when navigating entrenched doxic practices within professional soccer. While some of these practices may not align with current scientific evidence, attempting to challenge or overturn them in a confrontational manner can prove counterproductive. Abrasive approaches risk undermining the practitioners’ credibility and damaging relationships between players and staff, ultimately hindering their efforts to promote meaningful behavioural change. Such unintended consequences can arise when evidence-based recommendations are enforced without sensitivity to the practical and cultural realities of the soccer environment:

**[Participant 6, Sports Scientist]** I think that’s really important, that the way not to do it is to be abrasive. Like, for example, people have taken caffeine away in the past to not reduce the sensitivity when it comes to gaming. But in reality, that’s probably a bad thing, because you’re just going to cause arguments with the players, arguments with the staff, the players are probably going to end up getting caffeine from somewhere anyway. So even though scientifically it’s probably the right thing to do, I think all the barriers and all the arguments that causes along the way, it just isn’t worth it.”

In summary, the professional soccer environment operates as a hierarchical social field, defined by its own internal logic, power dynamics and entrenched cultural practices. Within this structure, coaches typically occupy dominant positions, exerting considerable influence over team operations and shaping players’ dietary behaviours. This aligns with previous research [3, 9] which highlights the tendency of coaches to intervene in nutritional practices through the imposition of dietary restrictions or prohibitions on certain foods, thereby overriding evidence-based nutritional strategies implemented by practitioners. Consequently, a nuanced understanding of coaches’ perspectives on nutrition and the ability to cultivate their support is essential for performance nutritionists seeking to implement effective interventions within elite soccer settings. Given their elevated status of the players, the communication approach adopted by nutritionists is important. Direct communication styles may provoke resistance and ultimately lead to disengagement from nutritional support. This was reflected in recent research, where professional players identified a lack of individual recognition as a key factor contributing to their disengagement with performance staff [38]. Moreover, practitioners must navigate the pervasive influence of doxa, the taken-for-granted norms that are deeply embedded within the professional soccer culture. Efforts to challenge or reform these practices, if done insensitively or without cultural awareness, may undermine practitioner credibility and hinder the likelihood of behavioural change. Previous research has emphasised that having a high level of contextual awareness within the sporting environment enables performance support team members to foster meaningful relationships and engage more effectively within their roles [39]. As such, the ability to operate effectively within this complex environment requires more than technical expertise; it demands the accumulation and deployment of key forms of capital, which are explored in the following theme.

### Capital: A Successful Performance Nutritionist Must Have Sufficient Technical Knowledge (Cultural Capital) But Also the Ability to Build Relationships (Social Capital)

Successfully navigating the complex and hierarchical field of professional soccer requires performance nutritionists to possess and strategically employ various forms of capital, a concept defined by Bourdieu as the resources individuals draw upon to maintain their position within a given social field [40]. Drawing on insights from a variety of clubs, key stakeholders in the present study identified cultural capital, which encompasses field-specific knowledge, qualifications and understanding of norms embedded within the environment [35], as particularly important. For instance, Participant 14 (coach) highlighted that “*sports nutrition knowledge first of all is probably the most important part of the game because you have to have the knowledge to back up the passion.*” In addition to sports nutrition expertise, familiarity with the physical and contextual demands of the sport itself was viewed as critical to establishing practitioner credibility, as articulated by Participant 13 (coach):

“You want to have an understanding of exactly what it takes at that level. And I’m not saying everyone must have played but having an understanding of what fuelling your body really looks like under some stress or under some physical exertion. It’s not the be all and end all, but it would help just so you can then pass on your processes.”

Interdisciplinary knowledge was also identified as a key asset for nutritionists operating within professional soccer clubs. Participants emphasised the value of having a broad understanding of other disciplines to strengthen the delivery of nutritional support. For instance, Participant 4 (sports scientist) explained:

“I think it’s good to have a general understanding of all the practices within the club, just to try and help push that nutritional support further. For example, if you are giving the lads a supplement or telling them that they can only have X amount of food. If you can provide that with the context of GPS metrics or strength metrics from the gym to actually show your understanding of why you’re putting forward the X plan.”

A lack of interdisciplinary awareness, particularly regarding the physical and tactical demands placed on the players, was perceived as potentially harmful. Misinterpretation of data or failure to account for the broader performance context could result in inappropriate nutritional interventions and lead to player mistrust. Participant 4 (sports scientist) further elaborated:

“The main things I could probably think of when nutritionists don’t have a complete understanding of the context of the game or the current situation of the player, whether it’s injury or whatever. I think it can be quite dangerous if they receive the wrong information. For example, if they don’t have a true understanding of a GPS report or a gym session and then try to find an intervention based on that. It could have consequences if it’s not discussed beforehand. Just off the top of my head I’m thinking if the player’s not running a set distance and it gets blamed on, they’re not fuelled correctly. Without having an understanding of the tactical element of the game it not being purely physical I think is important.”

Moreover, Participant 5 (physiotherapist) highlighted the need for nutritionists to maintain close alignment with rehabilitation processes, noting:

“Performance nutritionists should have a very strong finger on the pulse of rehabilitation. For example, you have to be very close to the rehabilitation so that can be achieved in a few different ways.”

In a similar manner, interdisciplinary awareness was also valued by non-performance staff. Participant 1 (chef) expressed appreciation for nutritionists who recognise the logistical and operational complexities of food service within a high-performance environment:

“From my side, it’s about teaching the team to understand what you need and me explaining to you there are parameters in some respects, not many. Timing is about it and ingredients that are hard to get at certain times in season. What I think would be good for us all to move forward is that we either do a small hour or an hour trying to explain to my team what you have to go through a day so that they understand.”

Social capital refers to the actual or potential resources accessible through a durable network of institutionalised relationships of mutual recognition and trust [14]. Within a professional soccer field, the ability to establish strong professional relationships with players was consistently identified by key stakeholders as a crucial asset. For instance, Participant 3 (coach) emphasised the importance of interpersonal competence alongside technical knowledge:

“A lot of people think it’s only the skill that matters but what matters is also the human that is performing the skill and it’s important that we understand the technicality of things. The nutritionist has to know about food, about atoms, about other stuffs. But he/she needs also to know how to communicate. He/she needs to know how to relate with people. He/she should know there’s different types of personalities, how can you get better to one, to others. Some people like a lot of talks, some like less talks, some like harder, some like stricter, some like softer.”

Participant 9 (coach) echoed this sentiment, stating that “*the role in my opinion needs to be someone that can have a relationship with players”.* Similarly, Participant 4 (sports scientist) acknowledged the importance of relationality while warning against becoming overly familiar:

“I think the two most important qualities are ability to build rapport and build good relationships with players. Not so much fanboying and being too friendly with them but being able to develop a relationship where they trust you, but you can also challenge them and push them to be better.”

These insights highlight the balance required in performance support roles, developing trust and connection without compromising authority or professionalism. In line with this, maintaining a high level of professional integrity was highly valued by key stakeholders. Participant 7 (medical doctor) outlines how attempts to please players at the expense of professional standards could undermine medical credibility:

“There have been colleagues in the past that were guilty of trying to please the players and that really undermines that person and their ethos as a medical team, particularly individuals, sneaking players, sugary foods for instance and it’s just, it really is undermining and it’s bad practice. It’s unprofessional because it’s sneaky behaviour and you can’t function as an entity like that.”

In summary, the effective delivery of nutrition support within the complex and hierarchical field of professional soccer requires the strategic application of multiple forms of capital. Of particular importance is cultural capital, encompassing sports nutrition knowledge and a nuanced understanding of both sports and soccer expertise. Stakeholders identified these attributes as essential for performance nutritionists to establish credibility within the field. Indeed, possessing the requisite knowledge and skill set is recognised as a fundamental requirement for members of a performance support team [39], whereas a lack of scientific rigour and failure to adopt evidence-based practices were previously identified as hallmarks for ineffective practitioners [11]. Moreover, interdisciplinary knowledge spanning sports science, rehabilitation and food service operations was regarded as critical for facilitating effective collaboration between performance nutritionists and other members of multidisciplinary support team. The importance of such integration is reinforced in a recent commentary, which cautions that siloed nutrition practice, responding only to the queries of individual practitioners, can be detrimental to collective team functioning and performance outcomes [2]. Understanding other people’s roles and responsibilities is therefore an antecedent for collaborative interdisciplinary teamwork [39]. From an applied educational standpoint, these findings highlight the need for programmes accredited by professional bodies (such as the UK Sport and Exercise Nutrition Register [SENr] or the Chartered Association of Sport and Exercise Sciences [CASES]) to transcend a focus on narrow technical competencies. There may be value in integrating relevant disciplines (e.g. sports science, physiology, psychology and physiotherapy) and applying sports nutrition within multi-disciplinary settings, thereby better equipping practitioners to navigate the complexities of elite sport environments, albeit how this integration occurs needs further consideration.

Equally vital was the development of social capital through the cultivation of strong professional relationships. Stakeholders strongly emphasised the importance of interpersonal skills, effective communication, and the ability to build rapport with the players and staff. This aligns with previous findings, which suggest that while it is relatively common to find practitioners with high levels of technical expertise, it is far less common to find those who also possess a deep appreciation for the sports’ unique culture and are capable of building relationship with players and coaches [15]. Indeed, the ability to assess, adapt and align to the environment was identified as a key attribute for good practice delivery in sports science and medicine support [41]. Furthermore, other research has highlighted the importance of embedding within the broader support team, particularly for practitioners who have yet to establish a strong rapport with players as a means of enhancing recognition and impact [42]. Maintaining professional boundaries and integrity was also regarded as essential, as efforts to overly accommodate players could compromise both credibility and cohesion within the medical and performance team. Effective practice in sports science and medicine were characterised by providing support within one’ scope of expertise and professional boundaries, which was viewed as fundamental in building trust among stakeholders [41]. In contrast, a poor relationship with colleagues has previously been identified as a hallmark of an ineffective performance nutrition practice [11]. Together, these insights underline the multi-faceted capital required of nutritionists to integrate effectively within elite soccer environments and the accumulation of capital are likely dependent on the habitus of the performance nutritionists.

### Habitus: The Success of a Nutritionist is Dependent on Their Ability to Accumulate Capital and is Shaped by Their Habitus

Habitus refers to the embodied dispositions shaped by an individual’s past experiences and social structures, which influence their perceptions, actions and decisions within a given field [12]. It represents the internalising ways of thinking, feeling and behaving that guide practice by shaping what individuals perceive as possible or appropriate [13]. In this sense, a practitioner’s actions are influenced not only by their current position within the professional soccer field but also by their social trajectory, which frames their capacity to recognise and respond to opportunities [13]. Accordingly, for performance nutritionists, possessing a habitus aligned with the norms and expectations of professional soccer was considered essential for acquiring the forms of capital that are deemed necessary to gain credibility and effectively navigate this complex environment.

Reflecting the broader cultural influences encountered throughout their careers, participants consistently emphasised the value attributed to passion and proactive engagement. For example, Participant 14 (coach) noted that *“the best nutritionists I’ve worked under are really passionate about their players being in the right condition or getting the right nutrients and the right food.”* Similarly, Participant 8 (player) described his willingness to work with nutritionists who show genuine interest in his well-being and health:

“When you feel like somebody’s passionate about what they’re doing, you want to work with them, you want to because you know they will go above and beyond to make sure, not even just for you but for themselves, to be the best that they can be.”

Conversely, Participant 9 (coach) identified a lack of initiative as a negative trait, criticising nutritionists who *“lack that engagement with the players. Not proactive and not giving them information and providing them with the stuff. Because if you expect them to do it, they won’t do it. You have to be on them.”*

Adaptability and flexibility emerged as essential attributes when working with key stakeholders in elite soccer environments. The ability to adjust one’s approach to accommodate the preferences, personalities and needs of others was consistently highlighted as central to building and sustaining productive relationships with both staff and players. Participant 5 (physiotherapist) stressed the importance of avoiding a rigid or uncompromising stance, particularly within a high-performance setting characterised by strong personalities:

“I think someone that isn’t flexible, someone that has got very strong, unflexible views will end up having a problem. It’s an environment where you’ve got a lot of alpha males. So, if you’re too strong in your opinions and too inflexible, I think that could end up spoiling the relationships that you could build with staff and players.”

Similarly, Participant 10 (player) emphasised the importance of adapting the nutritional plan based on feedback of the players, noting that dietary plans must be tailored to the unique preferences and tolerances of each player:

“Let’s say it is when the nutritionist doesn’t listen to the player and the feedback that the player gives. Because like I said, everyone is different so if a player doesn’t or cannot eat one type of a meal. But if a nutritionist doesn’t listen, basically saying that no, it’s good for you so you have to. Well, one thing will happen. The player won’t eat it, so he won’t have the same results. So like I said, it’s important to be able to personalise every single meal and every single diet of the player.”

Participant 11 (player) echoed this sentiment, cautioning against a “one-size-fits-all” approach, [43] which he described as potentially *“completely unattainable for some players”*. Participant 2 (physiotherapist) further reinforced this theme, highlighting that relationship building depends on the ability to find solutions that balance performance goals with individual preferences:

“How do you as an individual help that player believe what you’re doing is in their best interest and also make it tangible for them? So if you turn around and told me that I can no longer eat my favourite food ever, then automatically I’m switched off. If you tell me, “Your favourite food is really not good for you and these reasons why”, we can find a way to work it in for you—maybe you can have it once a fortnight as a treat, based on these other things happening. Then, all the sudden, I’m a bit more interested. Especially if you then give me alternatives that I also like.”

When individualising support where the players’ specific needs are essential, it may also place additional time (perhaps unrealistic) demands on practitioners. These demands can contribute to the risk of occupational fatigue, which is reflected in a recent survey showing that 42% of the medical and performance support staff working in the professional soccer were at risk of burnout [43]. In elite sport, occupational burnout has been associated with disengagement, reduced motivation, heightened emotional responses, withdrawal, impaired concentration and diminished disciplines, all of which can adversely affect support staff performance [44]. Consequently, resilience emerged as a critical attribute for practitioners. As participant 7 (medical doctor) reflected, *“I think you have to be very resilient, the season’s long, there’s ups and downs, there’s travelling, long hours, so I think you’ve got a lot of resilience and with that being a good team member.”* The capacity to manage mental fatigue and maintain a positive outlook was also highlighted by Participant 12 (player), who valued the influence of a practitioner’s demeanour on the wider group:

“If I see you coming in every day happy to be here and doing your best, it helps everyone. But if you’re sad, angry and don’t want to be here, that is the worst thing. You can be as smart as you want, but if you have bad energy, it affects everything. It is important to be part of the group, to help people and be there for them.”

In summary, the perceived effectiveness of performance nutritionists in elite soccer appears closely linked to their habitus, which refers to the internal dispositions shaped by prior experiences and social structures that align with the cultural norms of the professional game. Passion and proactive engagement were consistently valued by players and staff, while disengagement and a lack of initiative were perceived negatively. This aligns with previous research demonstrating that professional soccer players place high importance on genuine passion and effort from the performance staff [38]. Players value practitioners who demonstrate authentic care and prioritise their best interests [38]. Such compassion can elicit positive emotions and support healthy psychophysiological functioning, increasing players’ openness to opportunities and facilitating more favourable performance outcomes [45]. Coaches similarly identified a willingness to go above and beyond as a key marker of practitioner effectiveness [15]. Yet, this expectation reflects the broader doxa of soccer, where credibility is earned not only through expertise but through visible dedication. While such disposition can build trust and symbolic capital, they also risk normalising working long hours and burnout, highlighting the delicate balance practitioners must strike between cultural alignment, professional authority and sustainable practice. Furthermore, adaptability and flexibility also emerged as critical attributes, enabling practitioners to tailor nutritional strategies to the unique preferences, personalities and needs of stakeholders, thereby avoiding the pitfalls of a one-size-fits-all approach. This relational adaptability was underpinned by the capacity to balance performance goals with individual preferences, fostering greater adherence and rapport. Indeed, based on previous research, 80% of the respondents identified the ability to flex (adapt) communication styles as the most important trait of performance nutritionists [11], while versatility in navigating fast-paced environments and managing diverse and challenging personalities is also highly valued among performance support staff [39]. Moreover, resilience was identified as essential for sustaining performance in a demanding environment characterised by long seasons, travel and high pressure. The ability to manage mental fatigue and maintain a positive energising presence was considered integral not only to personal effectiveness but also to enhancing the collective environment.

Within elite sport, the demands of long working hours, high workload and frequent travel have consistently been identified as significant organisational stressors [46]. Such pressures can give rise to frustration and negative emotional responses, which, if left unmanaged, may extend beyond the individual and permeate the broader support team [46]. This spillover effect has the potential to create additional stress for colleagues, diminish morale and ultimately compromise the overall quality of work within the organisation [46]. Conversely, maintaining a positive presence is highly valued by elite athletes, as it not only prevents the transfer of stress onto them but also offers an important source of emotional support [47]. However, positioning constant positivity and resilience as expectations risks normalising unsustainable work practices and conflicts with the standard 37-h work week outlined by human resources in professional soccer. Within the cultural doxa of soccer where working “above and beyond” is valued, such expectations can obscure structural issues including unsociable hours and inadequate organisational support, by placing the burden on individuals to absorb an excessive workload and emotional strain. While this accrues symbolic capital for those perceived as highly dedicated, it simultaneously heightens the risk of burnout and undermines the long-term sustainability of practitioner well-being and service quality. Consequently, it is critical to consider how those with greater capital and influence (e.g. sporting directors, performance directors, head of departments), together with governing and regulatory bodies can enact systemic changes to challenge this entrenched culture and safeguard practitioner welfare.

## Practical Implications and Future Research Directions

Drawing on Bourdieu’s concepts of habitus, capital and field, this study identified the written and unwritten rules shaping the success of performance nutritionists in professional soccer from the perspectives of the key stakeholders (Fig. 1). The written rule centres on building strong technical foundations via academic qualifications and accreditation through professional bodies. Within the UK, this is typically achieved through registration with the SENr pathway: completing a relevant undergraduate degree (e.g. nutrition, dietetics, or sports and exercise science), followed by an SENr-accredited postgraduate degree for graduate registration and ultimately progressing to practitioner registration through applied experience and a competency-based portfolio [11].

The unwritten rules, however, extend beyond qualifications and technical expertise, requiring practitioners to navigate the social field of elite soccer by understanding the existing power dynamics, mobilising cultural and social capital, building trust with stakeholders and embodying a habitus aligned with the values of passion, adaptability, positivity and resilience. To prepare for this, neophyte nutritionists should be encouraged to deliberately cultivate interpersonal skills, particularly the ability to build trust and credibility with players, coaches and wider multi-disciplinary staff. Internships and structured work placements provide valuable platforms for developing these competencies [48], especially when supported by experienced and appropriately qualified supervisors who can scaffold practice and help acquire the essential social capital for effective practice [49]. Beyond entry-level training, hybrid models that embed practitioners within professional sport organisations such as research practitioner roles [50] and professional doctorate programmes [51] offer promising pathways to advance scientific knowledge while strengthening the applied impact. Crucially, when transitioning into new sporting environments, performance nutritionists must recognise and adapt to prevailing power dynamics, informal hierarchies and doxic practices, tailoring interventions aligned with the realities of the club environment.

While neophyte nutritionists must navigate both written and unwritten rules to accumulate symbolic capital, these dynamics can sometimes reinforce established practices. Entering the field with limited capital, junior practitioners may feel hesitant to question prevailing norms such as body composition targets that have been debated in the literature [3, 9] for fear of losing credibility or affecting employment. Although not reported by participants in this study, it is possible to see how such conditions may silence critical voices and perpetuate practices that diverge from best practice recommendations [52]. Furthermore, the emphasis of the habitus traits such as unwavering positivity and consistently working “above and beyond” is often regarded as a pre-requisite and may even be perceived as a means of accruing capital in the field. From a critical perspective, sustaining such workloads over time is unrealistic, which may in turn create pressures that affect well-being and service quality [43]. These challenges are not unique to nutritionists; evidence of burnout and compromised mental health has also been reported among wider performance support staff, coaches and managers in elite soccer settings [43, 53]. Given that these insights reflect the experiences of players across a variety of clubs, addressing these cultural issues likely requires organisational interventions rather than placing responsibility solely with the individual. Those with greater institutional and symbolic capital (e.g. sporting directors, performance directors) may be positioned as cultural architects capable of reshaping norms and beliefs within the field while mitigating the hierarchical pressures. Practical strategies may include implementing realistic workload expectations, providing adequate support and resources and fostering a culture that values a work-life balance [54]. The effectiveness of these approaches is closely linked to sufficient staffing as performance nutrition departments are often limited in resources [55]. Governing bodies such as SENr and the EPL may influence nutrition culture by establishing minimum standards, monitoring compliance and prioritising well-being alongside performance. While deeply entrenched norms in elite sport mean changes are likely to be gradual, sustained systemic support could progressively promote sustainable practices that prioritise practitioner welfare and strengthen the long-term effectiveness of performance nutrition provision.

While the insights presented here are derived from stakeholders at a single EPL club and thus may have limited generalisability, the themes identified drew upon their gamut of experiences and provide actionable considerations for both practitioners and institutions. Indeed, the stakeholders had previously played or coached across multiple other leagues, including EPL, Serie A, Bundesliga, Ligue 1, Turkish Süper Lig, Australian A-League, Scottish Premiership and Liga Portugal, bringing with them a breadth of diverse perspectives. Furthermore, all interviews were conducted by the club nutritionist. This insider position provided positive contributions in terms of access and a shared terminology/language but may have influenced participants’ openness because of the pre-existing professional relationships between the interviewer and interviewees. To mitigate potential bias, neutral and open questions were asked, whilst critical friends were incorporated into the data analysis process to support reflexive considerations. Together these processes enhance the trustworthiness of the findings. To strengthen this evidence base, future research should extend this work by engaging key stakeholders from multiple clubs, leagues and sports to establish a broader understanding of performance nutrition practice. Whilst this work has identified the importance of practitioners’ developing cultural and social capital alongside the performance habitus, there is now a need to explore how nutritionists can develop micropolitical, intrapersonal, and interpersonal knowledge and skills to work effectively with stakeholders (e.g. management, medical team and players). This work could involve advancing professional accreditation, devising educational interventions (e.g. experiential learning), implementing mentoring programmes and the development of applied case studies to support nutritionists develop as contextually influential professionals rather than technicians. Nevertheless, the findings presented here provide a practical lens through which readers can reflect and assess if the findings resonate with their experiences, the specific setting they operate in and the people they interact with [31].

## Conclusions

Our study identified three interrelated themes that characterise the attributes of a successful performance nutritionist within the EPL from the perspectives of key stakeholders. First, the professional soccer environment operates as a hierarchical social field, shaped by the authority of coaches and managers, the elevated status of the players and entrenched cultural norms and beliefs. To be effective within this setting, performance nutritionists must recognise and adapt to these implicit “rules of the game” through the strategic use of capital, including cultural capital (technical, sports specific and interdisciplinary knowledge) and social capital (ability to build relationship and trust with key stakeholders), while maintaining a high level of professional integrity. The capacity to accumulate and mobilise these forms of capital is underpinned by a habitus characterised by passion, adaptability, resilience and positivity, which resonates with the cultural expectations of professional soccer. Yet, these implicit “rules of the game” can constrain autonomy by reinforcing the doxic authority of coaches and valorising a habitus of overwork, which could be associated with burnout and reduced service quality. Sustaining practitioner effectiveness in the professional soccer field therefore requires not only technical and interpersonal skill development but also structural support from leaders in the club and governing bodies to reshape field norms and nutrition culture to advance practitioner welfare and the long-term impact of performance nutrition support.
